# Depression and anxiety in childhood-onset systemic lupus erythematosus: prevalence, associated factors, and impact on quality of life and family

**DOI:** 10.1186/s12969-025-01067-6

**Published:** 2025-02-11

**Authors:** Pitsamai Duangmala, Watchareewan Sontichai

**Affiliations:** 1 Department of Pediatrics, Faculty of Medicine, Chiang Mai University, Chiang Mai, Thailand; 2 Division of Rheumatology, Department of Pediatrics, Faculty of Medicine, Chiang Mai University, Chiang Mai, 50200 Thailand

**Keywords:** Anxiety, Depression, Family, Quality of life, Systemic lupus erythematosus

## Abstract

**Background:**

Depression and anxiety are common psychiatric manifestations in childhood-onset systemic lupus erythematosus (cSLE). This study aimed to determine the prevalence of clinically significant depression and anxiety, identify associated factors, and assess their impact on health-related quality of life (HRQOL) and family in patients with cSLE.

**Methods:**

We conducted a cross-sectional study of cSLE patients, aged 8–18 years. Patients completed the Children’s Depression Inventory (CDI), Screening for Child Anxiety Related Disorders (SCARED), Pediatric Quality of Life Inventory Generic Core Scale (PedsQL-GC), and Visual Analog Scale of pain intensity. Their parents completed the Pediatric Quality of Life Family Impact module (PedsQL family impact).

**Results:**

Of 91 patients, the median disease duration was 3.4 years (IQR 3.5), and the median SLE disease activity index 2000 score was 2 (IQR 6). The prevalence of clinically significant depression (CDI > 15) and clinically significant anxiety (SCARED ≥ 25) were 31.9% and 49.5%, respectively. Coexisting clinically significant depression and anxiety were found in 26 patients (28.6%). In multivariable analyses, older age at diagnosis was associated with clinically significant depression (OR 1.56, 95% CI 1.12–2.16, *p* = 0.008), while organ damage (OR 4.27, 95% CI: 1.19–15.31, *p* = 0.026) and pain score (OR 1.61, 95% CI: 1.11–2.32, *p* = 0.012) were associated with clinically significant anxiety. Patients with clinically significant depression or anxiety had significantly lower PedsQL-GC and PedsQL family impact scores compared to those without these symptoms.

**Conclusions:**

These results suggest that depression and anxiety are prevalent in cSLE and have negative impacts on HRQOL and family. Physicians should be aware of the presence of these psychological symptoms, particularly in patients with risk factors. Providing psychological counseling and prompt referral to psychiatrists could enhance HRQOL and family functioning.

## Background

Depression and anxiety disorders can develop in children at any age, from preschool through adolescence, and are common in the general population [1, 2] with increased prevalence among adolescents with chronic diseases [3], particularly those with chronic rheumatologic conditions [4]. Childhood-onset systemic lupus erythematosus (cSLE) is a multi-systemic autoimmune disease, with depression and anxiety disorders being common psychiatric manifestations. A limited number of studies have been carried out to evaluate the prevalence of clinically significant depression and anxiety in patients with cSLE and results show a wide range of prevalence rates, ranging from 6.7 to 59% for depressive symptoms and 34–37% for anxiety symptoms, respectively [5].

In adults with SLE, various factors, such as socioeconomic status, disease activity, organ damage, autoantibodies, and cumulative corticosteroid dose, have been associated with depressive and anxiety symptoms [6–10]. However, few studies have comprehensively explored these factors concerning depressive and anxiety symptoms among patients with cSLE, and the results are still inconclusive. This controversy partly arises from the difficulty in distinguishing whether depressive and anxiety symptoms in cSLE are a direct biological result of the disease or a psychological response to the challenges of living with a severe, chronic illness.

As in many chronic conditions, depression and anxiety disorders significantly impact daily life, affecting patients’ perceptions of their symptoms [9], medication adherence [11, 12], sleep quality [13], and health-related quality of life (HRQOL) [9, 14, 15] in children and adults with SLE. The negative impacts of depression and anxiety emerging in childhood and adolescence persist into young adulthood, including mental health, physical health, substance misuse, and poor education/employment status [16]. Thus, early identification of depression and anxiety disorders in children and adolescents with cSLE is important.

In addition, caring for a child with a chronic illness induces stress for the entire family, particularly the primary caregiver. An earlier study has demonstrated that relatives of children and adults with SLE exhibit impaired HRQOL compared to the general population and almost half of them suffer from symptoms of depression and anxiety [17]. Another study revealed that parental depression and children’s peer relationships had an impact on caregiver burden in a cohort of children and adolescents with cSLE [18]. To our knowledge, no studies specifically examined the effects of depression and anxiety on family functioning in cSLE.

This study aimed to determine the prevalence of clinically significant depression and anxiety in patients with cSLE, identify factors associated with these symptoms, and assess their impact on HRQOL and family in cSLE patients.

## Methods

### Patients

This cross-sectional study was conducted in the Pediatric Rheumatology and Nephrology clinicsat Chiang Mai University Hospital, Chiang Mai, Thailand from June 2022 to May 2023. The inclusion criteria were as follows: age between 8 and 18 years; diagnosis of cSLE based on the 2012 Systemic Lupus International Collaborating Clinics (SLICC) classification criteria [19] or the 2019 European League Against Rheumatism/ American College of Rheumatology (EULAR/ACR) classification criteria [20] before the eighteenth birthday; and a duration of illness longer than three months after the cSLE diagnosis to allow for an adjustment period. Patients were excluded, if they had intellectual disabilities, limited Thai proficiency, or difficulties in understanding and completing the questionnaires. All patients and their parents or caregivers provided written informed consent and assent before participation. The study protocol was approved by the Research Ethics Committee of the Faculty of Medicine, Chiang Mai University.

### Measures of depression and anxiety

We screened for clinically significant depression in patients with cSLE using the Children’s Depression Inventory (CDI) Version I. The CDI is a brief self-report questionnaire designed to assess depression in children and adolescents. It consists of 27 items, with each item having three choices corresponding to three levels of depressive symptoms. The total score ranges from 0 to 54, where a higher total score indicates a higher level of depressive symptoms. In this study, we utilized the Thai version of the CDI. A total score > 15 represents the presence of clinically significant depression, with demonstrated sensitivity and specificity above 79% and 91%, respectively, as shown in a previous cohort study of the Thai population [21].

We screened for clinically significant anxiety using the Thai version of Screening for Child Anxiety Related Disorders (SCARED). The SCARED is a self-report questionnaire that consists of 41 items, with each item having three choices [22]. The possible score ranges from 0 to 82, where a higher score indicates a higher level of anxiety symptoms. A total sum score of 25 or greater represents the presence of clinically significant anxiety [23].

### Measures of health-related quality of life and family impact

The Pediatric Quality of Life Inventory Generic Core Scale (PedsQL-GC) is a child self-report and parent proxy report tool, used to measure HRQOL in children and adolescents with health conditions [24]. The PedsQL-GC consists of 23 items, grouped into four domains: physical, emotional, social, and school function. Scores are expressed as percentages, with a higher score representing better HRQOL. The four domains can be divided into two summary scores; the physical health summary score and the psychosocial health summary score. The physical health summary score is represented by the physical functional score, while the psychosocial health summary score is represented by emotional, social, and school functional scores. A higher score indicates better HRQOL. The patient completed the Thai version of PedsQL-GC.

The Pediatric Quality of Life Family Impact Module (PedsQL™ 2.0 Family Impact Module) is a parent self-report tool, used to measure the impact of pediatric acute and chronic health conditions on parents and the family [25, 26]. It consists of 36 items, assessed parent functioning (physical functioning, emotional functioning, social functioning, cognitive functioning, communication and worry) and family functioning (daily activities and family relationships). Scores are expressed as percentages, with a higher score representing a lower negative impact. We utilized the Thai version of this questionnaire.

### Data collection

We collected demographic data of patients and their families including age, gender, parental marital status, monthly household income, highest household education level, and family history of psychiatric disorders. Clinical characteristics (age of diagnosis, duration of disease, obesity, presence of lupus nephritis, active skin lesion, laboratory data, disease activity, damage index, and current medications) were extracted from medical records. Patients were classified as obese if a body mass index-for-age z-score was more than two standard deviations above the median on the World Health Organization (WHO) growth standards [27]. Current prednisolone use was categorized into two groups: no use or low-dose (less than or equal to 10 mg daily prednisolone equivalent) and high-dose (more than 10 mg daily prednisolone equivalent). Patients completed the visual analog scale for pain, a self-report pain score ranging from 0 (no pain) to 10, with higher scores indicating higher pain levels.

Disease activity was measured by the Systemic Lupus Erythematosus Disease Activity Index 2000 (SLEDAI-2K) [28]. It comprises 24 items that include clinical and laboratory assessment at the time of the visit or in the proceeding 10 days. The total score ranged from 0 to 105, with a higher score indicating higher disease activity.

Accumulated organ damage in cSLE was determined by the SLICC/ACR damage index (SDI) [29]. Damage is defined as any nonreversible change for at least six months. The SDI consists of 41 items in 12 organ systems. The total score ranged from 0 (absence of damage) to 47, with a higher score indicating worsening organ damage.

### Statistical analysis

Data were analyzed using the SPSS 22.0 statistical software. The Shapiro-Wilk test was used for variable normality testing. Continuous variables were expressed as means and standard deviations (SD) or medians and interquartile ranges (IQR), and categorical variables were expressed as frequencies and percentages. Continuous variables were compared by using the Student’s t-test or Mann-Whitney U test as appropriate, while categorical variables were compared by using the chi-square/Fisher’s exact tests. The prevalence of clinically significant depression and anxiety in patients with cSLE was calculated. Spearman’s correlation analysis was used to investigate the relationship between parameters. A Spearman’s correlation coefficient (r) between 0.2 and 0.39 was considered weak, between 0.4 and 0.59 was considered moderate, between 0.6 and 0.79 was strong, and between 0.8 and 1.00 was considered very strong [30]. We analyzed factors associated with clinically significant depression and anxiety using univariable and multivariable logistic regression analyses. A *p*-value less than 0.05 was considered as statistical significance.

## Results

### Demographics and disease characteristics

A total of 91 patients with cSLE were enrolled, with 79 (86.8%) being female. The mean age at cSLE diagnosis was 10.6 ± 2.7 years. The mean age of patients in the study was 14.4 ± 2.3 years, and the median disease duration was 3.4 (IQR 3.5) years. Among these, 73.6% were from families in which the parents were married and cohabiting, and 65.9% had the highest household education level of less than college. A self-reported household income below the average monthly household income in Thailand (approximately 30,000 baht) [31] was found in 78.0% of patients. Six patients (6.6%) with cSLE had a family history of psychiatric disorders. The median SLEDAI-2K score was 2 (IQR 6), ranging from 0 to 16. The median SDI score was 0 (IQR 1), ranging from 0 to 3. Organ damage was present in 28 patients (30.8%). The median pain score was 1 (IQR 3). The majority of patients (92.3%) were taking prednisolone and 89% of patients were taking hydroxychloroquine at the time of the study visit. The median daily dose of prednisolone at the time of study was 10 (IQR 20) mg. Demographics and disease characteristics are shown in Table 1.

**Table 1:** Demographic and disease characteristics of patients with cSLE according to depression and anxiety status

Characteristics	All patients (*n* = 91)	Depression (*n* = 29)	Non-depression (*n* = 62)	*P*-value	Anxiety (*n* = 45)	Non-anxiety (*n* = 46)	*P*-value
Female, n (%)^a^	79 (86.8)	25 (86.2)	54 (87.1)	1.000	42 (93.3)	37 (80.4)	0.119
Age at the study (years), mean ± SD	14.4 ± 2.3	15.4 ± 1.9	13.9 ± 2.4	**0.002**	14.7 ± 2.3	14.1 ± 2.4	0.238
Age at diagnosis (years), mean ± SD	10.6 ± 2.7	11.5 ± 2.7	10.3 ± 2.6	**0.046**	10.8 ± 2.7	10.5 ± 2.6	0.515
Duration of disease (years), median (IQR)	3.4 (3.5)	3.9 (4.4)	3.4 (3.3)	0.759	3.8 (4.1)	3.2 (3.1)	0.625
Parental marital status, n (%)				0.428			0.366
Married Divorced Separated Widowed	67 (73.6) 14 (15.4) 7 (7.7) 3 (3.3)	21 (72.4) 3 (10.3) 4 (13.8) 1 (3.4)	46 (74.2) 11 (17.7) 3 (4.8) 2 (3.2)		35 (77.8) 4 (8.9) 4 (8.9) 2 (4.4)	32 (69.6) 10 (21.7) 3 (6.5) 1 (2.2)	
Household income (baht/month), n (%)				0.204			0.060
< 15,000 15,000–29,999 30,000–49,999 ≥ 50,000	54 (59.3) 17 (18.7) 12 (13.2) 8 (8.8)	19 (65.5) 5 (17.2) 5 (17.2) 0 (0)	35 (56.5) 12 (19.4) 7 (11.3) 8 (12.9)		23 (51.1) 12 (26.7) 8 (17.8) 2 (4.4)	31 (67.4) 5 (10.9) 4 (8.7) 6 (13.0)	
Highest household education level, n (%)				0.372			0.884
Less than college College and above	60 (65.9) 31 (34.1)	21 (72.4) 8 (27.6)	39 (62.9) 23 (37.1)		30 (66.7) 15 (33.3)	30 (65.2) 16 (34.8)	
Family history of psychiatric disorders, n (%)^a^	6 (6.6)	3 (10.3)	3 (4.8)	0.379	2 (4.4)	4 (8.7)	0.349
Obesity, n (%)	11 (12.1)	5 (17.2)	6 (9.7)	0.302	6 (13.3)	5 (10.9)	0.718
Presence of lupus nephritis, n (%)	51 (56.0)	18 (62.1)	33(53.2)	0.428	23 (51.1)	28 (60.9)	0.348
Active skin lesion, n (%)	15 (16.5)	6 (20.7)	9 (14.5)	0.415	9 (20.2)	6 (13.0)	0.396
Low complement, n (%)^a^	6 (6.6)	1 (3.4)	5 (8.1)	0.660	1 (2.2)	5 (10.9)	0.203
Presence of antiphospholipid antibody (*n* = 88), n (%)	30 (34.1)	10 (37.0)	20 (32.8)	0.698	15 (35.7)	15 (32.6)	0.759
Elevated anti-ds DNA (*n* = 89), n (%)	42 (47.2)	12 (44.4)	30 (48.4)	0.732	19 (44.2)	23 (50.0)	0.583
SLEDAI-2K score, median (IQR)	2 (6)	4 (10)	2 (6)	0.080	4 (9)	2 (4)	**0.009**
SDI score, median (IQR)	0 (1)	0 (1)	0 (1)	0.647	0 (1)	0 (0)	**0.004**
Organ damage, n (%)	28 (30.8)	10 (34.5)	18 (29.0)	0.600	20 (44.4)	8 (17.4)	**0.005**
Pain score, median (IQR)	1 (3)	2 (4)	1 (2)	**0.007**	2 (3)	0 (1)	**< 0.001**
Presence of pain, n (%)	55 (60.4)	22 (75.9)	33 (53.2)	**0.040**	35 (77.8)	20 (43.5)	**0.001**
Current medications, n (%)							
Prednisolone^a^	84 (92.3)	26 (89.7)	58 (93.5)	0.675	43 (95.6)	41 (89.1)	0.434
Prednisolone > 10 mg/day	54 (59.3)	18 (62.1)	36 (58.1)	0.717	32 (71.1)	22 (47.8)	**0.024**
Hydroxychloroquine^a^	81 (89.0)	26 (89.7)	55 (88.7)	1.000	38 (84.4)	43 (93.5)	0.197
Mycophenolate	43 (47.3)	16 (55.2)	27 (43.5)	0.301	21 (46.7)	22 (47.8)	0.912
Azathioprine^a^	10 (11.0)	2 (6.9)	8 (12.9)	0.493	4 (8.9)	6 (13.0)	0.739
Methotrexate^a^	4 (4.4)	1 (3.4)	3 (4.8)	1.000	2 (44.4)	2 (43.5)	1.000
Cyclophosphamide^a^	3 (3.3)	1 (3.5)	2 (3.2)	1.000	3 (6.7)	0 (0)	0.117
Antihypertensive	51 (56.0)	16 (55.2)	35 (56.5)	0.909	26 (57.8)	25 (54.3)	0.742

The bold value indicates statistical significance at the 0.05 level. *SD* standard deviation, *IQR* interquartile range, *anti-ds DNA* anti double-stranded DNA, *SLEDAI-2K* Systemic Lupus Erythematosus Disease Activity Index 2000, *SDI* Systemic Lupus International Collaborating Clinics/American College of Rheumatology Damage Index. ^a^ Fisher’s exact test

### Prevalence of clinically significant depression and anxiety

The CDI score ranged from 0 to 44, with a total median score of 11 (IQR 12). The prevalence of clinically significant depression was observed in 29 patients (31.9%). The SCARED score ranged from 0 to 65, with a total median score of 24 (IQR 20). The prevalence of clinically significant anxiety was noted in 45 patients (49.5%). Among these patients, 26 patients (28.6%) had coexisting clinically significant depression and anxiety.

Table 1 demonstrates the comparison of demographic and disease features between patients with and without clinically significant depression, as well as those with and without clinically significant anxiety. Patients with clinically significant depression significantly were significantly older at the time of enrollment (15.4 ± 1.9 vs. 13.9 ± 2.4 years, *p* = 0.002) and at the time of diagnosis (11.5 ± 2.7 vs. 10.3 ± 2.6 years, *p* = 0.046) compared with those without clinically significant depression. Additionally, the median pain score was significantly higher in the group with clinically significant depression, compared with the non-depressive group [2 (IQR 4) vs. 1 (IQR 2), *p* = 0.007].

For anxiety, the median SLEDAI-2K score was significantly higher in patients with clinically significant anxiety, compared with those without anxiety [4 (IQR 9) vs. 2 (IQR 4), *p* = 0.009]. Similarly, the median pain score was significantly higher in the clinically significant anxiety group compared with the non-anxiety group [2 (IQR 3) vs. 0 (IQR 1), *p* < 0.001). Furthermore, we found significantly higher numbers of organ damage (44.4% vs. 17.4%, *p* = 0.005), and the current use of daily prednisolone of more than 10 mg (71.1% vs. 47.8%, *p* = 0.024) in patients with clinically significant anxiety, compared with those without anxiety.

### Factors associated with clinically significant depression and anxiety

Spearman’s correlation test showed a strong positive correlation between the CDI score and the SCARED score (*r* = 0.71, *p* < 0.001) as shown in Table 2. The CDI score showed a moderate correlation with the pain score (*r* = 0.44, *p* < 0.001) and a weak correlation with the SLEDAI-2 K score (*r* = 0.22, *p* = 0.037). As for anxiety, the SCARED score demonstrated a moderate correlation with the pain score (*r* = 0.41, *p* < 0.001) and weak correlations with both the SLEDAI-2K score (*r* = 0.21, *p* = 0.049) and SDI score (*r* = 0.27, *p* = 0.009).

**Table 2:** Correlations between depressive, anxiety, pain, disease activity, damage index, HRQOL, and family impact scores

Variables	CDI	SCARED	Pain score	SLEDAI-2K	SDI	PedsQL-GC	PedsQL family impact
**CDI**	1.00						
**SCARED**	0.71**	1.00					
**Pain score**	0.44**	0.41**	1.00				
**SLEDAI-2K**	0.22*	0.21*	0.26*	1.00			
**SDI**	0.12	0.27*	0.35**	0.25*	1.00		
**PedsQL-GC**	-0.65**	-0.76**	-0.41**	-0.22*	-0.25*	1.00	
**PedsQL family impact**	-0.38**	-0.35**	-0.23*	-0.19	-0.25*	0.48**	1.00

*CDI* Children’s Depression Inventory, *SCARED* Screening for Child Anxiety Related Disorders, *SLEDAI-2K* Systemic Lupus Erythematosus Disease Activity Index 2000, *SDI* Systemic Lupus International Collaborating Clinics/American College of Rheumatology Damage Index, *PedsQL-GC* Pediatric Quality of life Inventory Generic Core Scale, *PedsQL family impact* Pediatric Quality of Life Family Impact. *p-value < 0.05, **p-value < 0.01

In the regression model evaluating the association between factors and clinically significant depression, the univariable analysis revealed that older age at diagnosis (OR 1.20, 95% CI: 1.00-1.43, *p* = 0.047) and pain score (OR 1.30, 95% CI: 1.06–1.60, *p* = 0.013) were significantly associated with clinically significant depression. In multivariable logistic regression analysis with adjustment for confounding covariates, only older age at diagnosis was associated with clinically significant depression (OR 1.56, 95% CI: 1.12–2.16, *p* = 0.008). Results are shown in Table 3.

**Table 3:** Factors associated with clinically significant depression in patients with cSLE

Variable	Univariable analysis		Multivariable analysis	
	OR (95% CI)	*P*-value	OR (95% CI)	*P*-value
Gender	0.93 (0.26–3.37)	0.907	0.79 (0.16–3.84)	0.767
Age at diagnosis	**1.20 (1.00-1.43)**	**0.047**	**1.56 (1.12–2.16)**	**0.008**
Disease duration	1.04 (0.88–1.24)	0.637	1.34 (0.98–1.84)	0.071
Parental marital status	1.10 (0.41–2.96)	0.858	0.80 (0.22–2.85)	0.725
Family history of psychiatric disorders	2.27 (0.43-12.00)	0.335	2.51 (0.28–22.64)	0.413
Presence of lupus nephritis	1.44 (0.58–3.54)	0.429	1.88 (0.63–5.60)	0.258
Presence of antiphospholipid antibody	1.21 (0.47–3.11)	0.698	1.28 (0.37–4.39)	0.692
Elevated anti-ds DNA	0.85 (0.34–2.12)	0.732	0.88 (0.28–2.78)	0.824
SLEDAI-2K	1.10 (1.00-1.21)	0.059	1.11 (0.96–1.28)	0.162
Organ damage	1.29 (0.50–3.30)	0.600	0.74 (0.21–2.61)	0.644
Pain score	**1.30 (1.06–1.60)**	**0.013**	1.24 (0.93–1.67)	0.141
Prednisolone > 10 mg/day	1.18 (0.48–2.92)	0.717	0.78 (0.23–2.62)	0.683

The bold value indicates statistical significance at the 0.05 level. *OR* odds ratio, *CI* confidence interval, *anti-ds DNA* anti double-stranded DNA, *SLEDAI-2K* Systemic Lupus Erythematosus Disease Activity Index 2000

As for anxiety, SLEDAI-2K (OR 1.14, 95% CI: 1.03–1.26, *p* = 0.012), organ damage (OR 3.80, 95% CI: 1.45–9.95, *p* = 0.007), pain score (OR 1.63, 95% CI: 1.24–2.15, *p* < 0.001), and the current use of daily prednisolone more than 10 mg (OR 2.69, 95% CI: 1.13–6.39, *p* = 0.025) were associated with clinically significant anxiety in univariable analysis. Multivariable logistic regression analysis revealed that organ damage (OR 4.27, 95% CI: 1.19–15.31, *p* = 0.026) and pain score (OR 1.61, 95% CI: 1.11–2.32, *p* = 0.012) were independently associated with clinically significant anxiety (Table 4).

**Table 4:** Factors associated with clinically significant anxiety in patients with cSLE

Variable	Univariable analysis		Multivariable analysis	
	OR (95% CI)	*P*-value	OR (95% CI)	*P*-value
Gender	3.42 (0.86–13.53)	0.082	3.06 (0.51–18.40)	0.222
Age at diagnosis	1.05 (0.90–1.23)	0.510	1.16 (0.89–1.52)	0.264
Disease duration	1.03 (0.88–1.21)	0.692	1.08 (0.81–1.43)	0.611
Parental marital status	0.65 (0.25–1.68)	0.376	0.37 (0.09–1.46)	0.154
Family history of psychiatric disorders	0.49 (0.09–2.81)	0.422	0.16 (0.01–2.35)	0.181
Presence of lupus nephritis	0.67 (0.29–1.54)	0.349	0.71 (0.23–2.20)	0.554
Presence of antiphospholipid antibody	1.15 (0.48–2.77)	0.759	1.95 (0.52–7.28)	0.319
Elevated anti-ds DNA	0.79 (0.34–1.82)	0.583	0.50 (0.15–1.66)	0.256
SLEDAI-2K	**1.14 (1.03–1.26)**	**0.012**	1.10 (0.94–1.30)	0.231
Organ damage	**3.80 (1.45–9.95)**	**0.007**	**4.27 (1.19–15.31)**	**0.026**
Pain score	**1.63 (1.24–2.15)**	**< 0.001**	**1.61 (1.11–2.32)**	**0.012**
Prednisolone > 10 mg/day	**2.69 (1.13–6.39)**	**0.025**	2.56 (0.72–9.05)	0.146

The bold value indicates statistical significance at the 0.05 level. *OR* odds ratio, *CI* confidence interval, *anti-ds DNA* anti double-stranded DNA, *SLEDAI-2K* Systemic Lupus Erythematosus Disease Activity Index 2000

### Impact of clinically significant depression and anxiety on HRQOL and family

The results of the HRQOL and family impact in the subsets of patients are shown in Table 5. Overall, patients with clinically significant depression perceived their total HRQOL significantly lower than those without depression (58.8 ± 15.3 vs. 78.5 ± 13.4, *p* < 0.001). This pattern was also observed in the physical health, psychosocial health, emotional functioning, social functioning, and school functioning domains. Similarly, patients with clinically significant anxiety had significantly lower mean scores of PedsQL-GC, compared with those without anxiety, both overall (61.3 ± 14.1 vs. 83.0 ± 11.3, *p* < 0.001) and in all other domains.

**Table 5:** PedsQL-GC and PedsQL-family impact scores in various subgroups of patients with cSLE

Variable	All patients (*n* = 91)	Depression			Anxiety			
		Depression (*n* = 29)	Non-depression (*n* = 62)	*P*-value	Anxiety (*n* = 45)	Non-anxiety (*n* = 46)	*P*-value	
PedsQL-GC total score	72.2 ± 16.7	58.8 ± 15.3	78.5 ± 13.4	< 0.001	61.3 ± 14.1	83.0 ± 11.3	< 0.001	
Physical	73.4 ± 20.3	61.9 ± 20.9	78.8 ± 17.8	< 0.001	63.5 ± 19.7	83.1 ± 15.9	< 0.001	
Psychosocial	71.6 ± 17.1	57.2 ± 15.3	78.4 ± 13.4	< 0.001	60.1 ± 14.5	82.9 ± 10.8	< 0.001	
• Emotional	69.1 ± 21.2	49.8 ± 16.1	78.1 ± 16.8	< 0.001	55.2 ± 18.1	82.6 ± 14.0	< 0.001	
• Social	76.7 ± 20.7	61.9 ± 22.3	83.6 ± 15.9	< 0.001	65.9 ± 20.8	87.2 ± 14.3	< 0.001	
• School	69.1 ± 21.0	59.8 ± 21.7	73.5 ± 19.4	0.006	59.1 ± 23.3	78.9 ± 12.3	< 0.001	
PedsQL-family impact total score	77.9 ± 15.5	69.3 ± 16.3	81.9 ± 13.5	0.001	71.5 ± 15.8	84.2 ± 12.5	< 0.001	
Parent functioning	79.2 ± 15.9	70.2 ± 16.4	83.4 ± 13.9	< 0.001	72.7 ± 16.8	85.5 ± 12.2	< 0.001	
• Physical functioning	77.2 ± 19.6	69.7 ± 19.9	80.7 ± 18.6	0.015	71.7 ± 20.2	82.6 ± 17.6	0.007	
• Emotional functioning	76.2 ± 18.3	65.2 ± 17.0	81.4 ± 16.7	< 0.001	68.6 ± 16.9	83.7 ± 16.7	< 0.001	
• Social functioning	83.4 ± 21.0	74.4 ± 24.6	87.6 ± 17.8	0.013	75.3 ± 24.9	91.3 ± 12.2	< 0.001	
• Cognitive functioning	79.8 ± 19.3	71.4 ± 23.3	83.8 ± 15.8	0.013	75.2 ± 22.2	84.4 ± 14.7	0.024	
• Communication	80.9 ± 23.0	67.8 ± 28.4	86.6 ± 17.3	0.002	71.5 ± 25.7	89.5 ± 16.0	< 0.001	
• Worry	68.1 ± 22.4	61.2 ± 24.7	71.3 ± 20.6	0.062	60.2 ± 21.6	75.8 ± 20.5	0.001	
Family functioning	78.6 ± 19.7	69.4 ± 22.3	83.0 ± 16.9	0.006	72.9 ± 21.3	84.3 ± 16.4	0.005	
• Daily activities	72.4 ± 24.9	59.5 ± 28.2	78.5 ± 20.8	0.001	64.1 ± 27.6	80.6 ± 19.0	0.001	
• Family relationships	84.8 ± 18.0	79.3 ± 19.9	87.4 ± 16.6	0.063	81.7 ± 19.0	87.9 ± 16.7	0.098	

Values are presented as mean ± SD. *PedsQL-GC* Pediatric Quality of Life Inventory Generic Core Scale, *PedsQL family impact* Pediatric Quality of Life Family Impact

For the impact on family, the mean total scores of all patients were 77.9 ± 15.5, with the highest mean score reported for the family relationships domain (84.8 ± 18.0), and the lowest mean score reported for the worry domain (68.1 ± 22.4). The mean scores of patients with clinically significant depression were significantly lower than those without clinically significant depression in the total score (69.3 ± 16.3 vs. 82.0 ± 13.5, *p* = 0.001) and all subscales, except for the worry and family relationships domains. Additionally, significant differences were observed between the PedsQL-family impact total score in the anxiety group compared to the non-anxiety group (71.5 ± 15.8 vs. 84.2 ± 12.5, *p* < 0.001), as well as in all subscale scores, except for the family relationships domain.

As shown in Table 2, the CDI score had a strong negative correlation with the PedsQL-GC score (*r* = -0.65, *p* < 0.001) and a weak negative correlation with the PedsQL family impact total score (*r* = -0.38, *p* < 0.001). The SCARED score had a strong negative correlation with the PedsQL-GC score (*r* = -0.76, *p* < 0.001) and a weak negative correlation with the PedsQL family impact total score (*r* = -0.35, *p* = 0.001).

## Discussion

In this cross-sectional study, we found that 31.9% of patients with cSLE had symptoms indicating clinically significant depression and 49.5% of patients had symptoms indicating clinically significant anxiety. Among these patients, 28.6% had coexisting clinically significant depression and anxiety. Older age at diagnosis was associated with clinically significant depression, while organ damage and pain score were associated with clinically significant anxiety. Patients with clinically significant depression and/or anxiety reported lower HRQOL scores overall and in all domains compared to those without these psychiatric symptoms. We presented novel data on the impacts of clinically significant depression and anxiety on family including parent and family functioning.

Our study revealed clinically significant depression was prevalent in patients with cSLE. Previous studies in various countries have reported that 20-58.8% of children and adolescents with cSLE exhibited depressive symptoms [12, 15, 32–34]. About half of our study patients reported clinically significant anxiety, which was much higher than the 37–39% prevalence reported in prior studies [15, 32]. This high heterogeneity of prevalence values of depressive and anxiety symptoms among cSLE patients was attributed to multiple factors, including disease characteristics, the heterogeneity of the assessment’s scales, unclear definitions of depression and anxiety disorders used across studies, as well as cultural and social backgrounds.

Clinically significant depression and anxiety were highly correlated and coexisted in almost one-third of our patients. In comparison with studies conducted in different regions, the prevalence of coexisting clinically significant depression and anxiety was found to be higher among the Thai pediatric population. Previous studies reported comorbid depression and anxiety in 8–10% of pediatric patients with cSLE; however, one study focused on adolescents and young adults with cSLE aged 12–21 years [35] and another study included patients with cSLE and mixed connective tissue disease [36]. These findings suggest that depression and anxiety in this population are not isolated conditions. Instead, they frequently co-occur. This is an important point because patients with comorbid depressive and anxiety symptoms have poorer outcomes and a greater risk of suicide than either condition in isolation [16, 37, 38] as well as decreased response to treatment leading to different treatment approaches [37, 38].

Factors associated with depressive and anxiety symptoms in cSLE have been investigated, but the findings remain controversial. The difficulty in determining the origin of these psychological symptoms arises from to two possible causes: the biological effects of SLE (e.g. neuroinflammation) and a secondary, reactive response to the challenges of dealing with a severe and chronic disease (e.g. physical limitations, pain, uncertainty, or social stigma). These factors may also overlap, further complicating the understanding of their underlying causes.

We identified older age at diagnosis as being associated with clinically significant depression. This association could be explained by the observation that depressive levels tend to be low before puberty and increase from middle childhood through adolescence before stabilizing in early adulthood in the general population [39, 40]. In our study, patients with cSLE typically experienced disease onset during early adolescence (mean age 10.6 ± 2.7 years), which is a critical period in their psychosocial development involving independence, self-identity, and the acquisition of essential skills needed for a successful transition to adult roles.

Concerning organ damage, there are inconsistent findings regarding its association with these psychiatric symptoms. The present study revealed an association between organ damage and anxiety symptoms, in contrast to a previous study involving children and adolescents with cSLE and mixed connective tissue disease, which showed that organ damage was not associated with anxiety symptoms but tended to increase the risk of depressive symptoms [36]. Nevertheless, establishing a definite association is challenging due to the scarcity of studies investigating the association between organ damage and anxiety in cSLE to date.

In addition, we found that pain score had significant moderate correlations with depressive and anxiety symptoms and was an associated factor for clinically significant anxiety in patients with cSLE. These findings align with a previous study of adolescents and young adults with cSLE, which indicated a correlation between the severity of depressive and anxiety symptoms and increased pain severity [34]. In adults with SLE, several studies have also shown that pain is closely associated with depression and anxiety. Waldheim et al. indicated adults with SLE scoring higher degrees of pain (pain score 40–100 mm) were burdened with more anxiety and depression compared to patients with lower levels of pain (pain score 0–39 mm) [41]. A study involving Chinese adult patients with SLE also showed higher levels of pain predicted a higher risk of anxiety in a multivariable analysis [42]. It implies that enhancing pain management can not only relieve patients’ pain but also potentially reduce anxiety and depressive symptoms of patients.

A prior study has demonstrated that the HRQOL of patients with cSLE is poorer than that of healthy children [15]. Among various factors, depressive and anxiety symptoms were found to be correlated with a reduced HRQOL score [15]. Consistent with this previous study, our study also revealed that patients with clinically significant depression and/or anxiety had lower PedsQL-GC scores overall and in all domains compared to those without these symptoms. Moreover, the depressive and anxiety scores were significantly negatively correlated with the HRQOL score with strong correlations. Donnelly et al. reported that cSLE patients with clinically significant depressive symptoms at the initial visit had poorer HRQOL at the six-month follow-up [43].

In the present study, we also analyzed the impact of clinically significant depression and anxiety on family. Notably, we found a considerable impact on family including parent and family functioning among cSLE patients with the presence of clinically significant depressive and/or anxiety symptoms. Results from parents’ reports on their own HRQOL in the group with clinically significant depression and/or anxiety were significantly worse than those reported by parents of patients without these symptoms. To date, there is a lack of data to assess the association of depression and anxiety with parent and family functioning in the pediatric population with cSLE. One study from Zeng et al. revealed that family members of children and adults with SLE who experienced more severe symptoms of depression or anxiety had a worse HRQOL compared to those with less depression or anxiety. However, they did not investigate the association between the depressive and anxiety status of patients and the quality of life of their relatives [17]. In terms of family functioning, our patients with clinically significant depression and/or anxiety had lower scores in the daily activities domain compared to those without symptoms. Although there was a trend indicating that the scores in the family relationships domain among patients with clinically significant depressive and/or anxiety symptoms were lower than those without symptoms, it did not reach statistical significance.

Our study offers important contributions to the knowledge base on depression and anxiety in children and adolescents with cSLE. We evaluated potential associated factors including socioeconomic status, disease activity, organ damage, pain intensity, presence of autoantibody, and steroid treatment. We also provided new insights into the effects of depression and anxiety on the HRQOL of patients and parents as well as family functioning within this specific population. However, there are several limitations to our study. First, we did not use a disease-specific questionnaire to probe HRQOL domains relevant to children with rheumatic diseases, which may limit a valid and reliable determination of HRQOL. Second, a possible limitation when using self-reported questionnaires is the risk of overestimation of the prevalence of depression and anxiety disorder [32]. Although these questionnaires are highly accurate for diagnosing depression and anxiety disorder, additional psychiatric interviews could provide a more accurate diagnosis. Third, due to our small sample size, we were limited in our power to detect associations. Last, our study is a cross-sectional study that assessed patients at only a single point. Our findings can only indicate associations and point out trends but cannot prove causation. Longitudinal studies with larger sample sizes are therefore needed to confirm and further explore the findings.

## Conclusions

In summary, the current study stressed the high prevalence of clinically significant depression and anxiety among children and adolescents with cSLE. Multivariable analyses showed older age at the diagnosis was significantly associated with the presence of clinically significant depression while organ damage and higher pain score were associated with the presence of clinically significant anxiety. Patients with clinically significant depression and/or anxiety had poorer HRQOL than those without these psychological symptoms. Our findings provide new insight into the relationship between depression and anxiety in the family of patients with cSLE. Based on these findings, we suggest for clinical practice that regular screening for these psychological symptoms in children and adolescents with cSLE, especially high-risk patients, should be a standard part of routine care. Psychological counseling and prompt referral to psychiatrists may help improve HRQOL and family functioning.

## Data Availability

No data other than what is included in this article will be shared.

## Abbreviations

cSLE: Childhood-onset systemic lupus erythematosus
HRQOL: Health-related quality of life
SLICC: Systemic lupus international collaborating clinics
EULAR: European league against rheumatism
ACR: American college of rheumatology
CDI: Children’s depression inventory
SCARED: Screening for child anxiety related disorders
PedsQL-GC: Pediatric quality of life inventory generic core Scale
WHO: World health organization
SLEDAI-2K: Systemic lupus erythematosus disease activity index 2000
SDI: SLICC/ACR damage index
SD: Standard deviation
IQR: Interquartile range
